# Association Between Rare Earth Element Cerium and the Risk of Oral Cancer: A Case-Control Study in Southeast China

**DOI:** 10.3389/fpubh.2021.647120

**Published:** 2021-05-25

**Authors:** Baochang He, Jing Wang, Jing Lin, Jinfa Chen, Zhaocheng Zhuang, Yihong Hong, Lingjun Yan, Lisong Lin, Bin Shi, Yu Qiu, Lizhen Pan, Xiaoyan Zheng, Fengqiong Liu, Fa Chen

**Affiliations:** ^1^Department of Epidemiology and Health Statistics, School of Public Health, Fujian Medical University, Fuzhou, China; ^2^Key Laboratory of Ministry of Education for Gastrointestinal Cancer, Fujian Medical University, Fuzhou, China; ^3^Department of Oral and Maxillofacial Surgery, The First Affiliated Hospital of Fujian Medical University, Fuzhou, China; ^4^Laboratory Center, School of Public Health, Fujian Medical University, Fuzhou, China

**Keywords:** rare earth elements, cerium, oral cancer, inductively coupled plasma mass spectrometry, risk assessment

## Abstract

Cerium (Ce), the most abundant of rare earth elements in the earth's crust, has received much health concerns due to its wide application in industry, agriculture, and medicine. The current study aims to evaluate whether there is an association between Ce exposures and the risk of developing oral cancer. Serum Ce level of 324 oral cancer patients and 650 matched healthy controls were measured by inductively coupled plasma mass spectrometry. Association between Ce level and the risk of oral cancer was estimated with an unconditional logistic regression model. Serum Ce concentrations in the oral cancer patients and controls were 0.57 (0.21–3.02) μg/L and 2.27 (0.72–4.26) μg/L, respectively. High level of Ce was associated with a decreased risk of oral cancer (OR: 0.60, 95% CI: 0.43–0.84). Stronger inverse associations between high level of Ce and oral cancer risk were observed among those with smoking (OR: 0.46, 95% CI: 0.27–0.79), drinking (OR: 0.50, 95% CI: 0.26–0.96), limited intake of leafy vegetables (OR: 0.40, 95% CI: 0.22–0.71) and fish (OR: 0.52, 95% CI: 0.33–0.83). There were significant multiplicative interactions between Ce level and alcohol drinking or intake of leafy vegetables and fish (all P_interaction_ <0.05). This preliminary case-control study suggests an inverse association between high serum Ce level and the risk of oral cancer. Further prospective studies with a larger sample size are needed to confirm the findings.

## Introduction

Oral cancer is a common malignant tumor on the oral and maxillofacial. An estimated 354,864 new cases and 177,384 deaths from lip and oral cancers occurred globally in 2018, accounting for 2.0 and 1.9% of all new cancer cases and deaths (1). To date, there have been many studies on the etiology of oral cancer, among which tobacco, alcohol, and limited intake of fish and vegetables are the main known risk factors for oral cancer (2, 3). However, oral cancer still occurs in those with non-smoking, non-drinking and dietary balance, suggesting that there are other unknown factors associated with oral cancer.

Cerium (Ce) is the rare earth elements with the highest abundance in earth's crust (4). It was widely used in the field of high technologies and traditional industries such as in agriculture and the production of phosphors, alloys and catalysis with its unique trivalent state (5). Ingestion of contaminated food and water, direct uptake via medical administration, and occupational exposure are the potential sources for the transfer of cerium from the environment to the human body posing health risks (6–8).

Numerous studies indicated that Ce played a vital role in biological processes relevant to various cancers. Ce has been reported to exhibit antioxidant activity by scavenging ROS in healthy cells while acting as a pro-oxidant and having anti-cancer activity in cancer cells by inducing ROS formation (9). *In vitro* experiments demonstrated that CeO_2_ nanoparticles induced significant oxidative stress leading to decreased viability of human lung cancer cells and colon cancer cells (10, 11). An *in vivo* study also revealed a new role of CeO_2_ nanoparticles as a novel anti-angiogenic agent that attenuated tumor growth in a preclinical mouse model of ovarian cancer (12). Madero-Visbal et al. also reported that CeO_2_ nanoparticles may decrease radiation-induced xerostomia and G-III dermatitis in head and neck cancer (13). However, most previous studies focused on cell or animal experiments. So far, limited data related to Ce and cancers are available in population-based on an epidemiological study, let alone the associations of Ce on oral cancer risk in southeastern China where a high yield of rare earth is produced. Therefore, the purpose of this study was (1) to measure the concentrations of Ce in the serum of oral cancer patients in comparison with the cancer-free controls; (2) to evaluate the association of serum levels of Ce with oral cancer risk.

## Methods

### Study Participants

This case-control study was conducted in Fujian province, China, from September 2011 to January 2018. A total of 461 oral cancer patients were recruited from the First Affiliated Hospital of Fujian Medical University. Cases met the following inclusion criteria: (1) all cases were primary oral cancer patients, diagnosed after histological confirmation; (2) all cases reside in Fujian Province; (3) all cases aged 20 to 80 years. The exclusion criteria of cases included: (1) recurrent or metastasized oral cancer; (2) those who undertook radiotherapy or chemotherapy; (3) any history of severe systemic diseases such as severe liver and kidney dysfunction and AIDS; (4) long-term use of any dietary supplements. This left 324 (72.3%) patients in the final cohort for analysis.

During the study period, a control group of 650 individuals was randomly selected from the physical examination center of the same hospital who had no history of cancers. The healthy status was identified based on the results of physical examination. Cases were frequency matched to controls, in a ratio of about 1:2, considering both age and sex. The exclusion criteria were as follows: (1) those who were not in the same period as the case group; (2) those who were not from single households; (3) those who did not reside in Fujian Province, or those aged <20 or >80 years; (4) those who used long-term dietary supplements.

This study was performed according to the ethical principles of the Declaration of Helsinki and approved by the Institutional Review Board of Fujian Medical University (Approval number: 2011053).

### Data Collection

Signed informed consents were provided from all participants. Data were collected through in-person interview using a standardized questionnaire which included the information about demographic characteristics, family history of cancer, smoking habits, alcohol consumption, as well as dietary intake. As for dietary data, the questionnaire contained five broad categories (red meat: pork/beef/lamb; fish; seafood: shrimp/crab/shellfish; eggs: chicken and duck eggs; leafy vegetables; dietary supplements). Then, participants were asked how often intake of each food item according to the following options: 3 times per day; 2 times per day; 1 time per day; 5–6 times per week, 3–4 times per week; 1–2 times per week; <1 time per week or not at all. And these questions about dietary habits were directed 1 year prior to cancer diagnosis or interview (for controls). The overall response rate of the questionnaire was 90.2% (96.1% for cases and 87.5% for controls). All participants were engaged in the study after being interviewed directly.

### Collection and Analysis of Blood Samples

Fasting venous blood samples (about 3–5 mL) for determining Ce levels of cases were collected on the second day of hospitalization prior to any drug treatment or examination. Then the control serum samples (about 3–5 mL) were from individuals seeking a routine health check up in the physical examination center. Then, all samples were centrifuged at 1,509 g for 10 min at 4°C, and serum was collected. Serum samples were immediately transferred and stored at −80 °C until needed for analysis.

Detailed laboratory methods for measurements had been described previously (14). Briefly, serum samples were digested by a microwave digestion system (PreeKem, China). Then, inductively coupled plasma massed spectrometry (ICP-MS, NexION 350X; Perkin-Elmer, USA) was used to determine serum Ce concentration. For the analytic quality control, human hair powders (GBW07601a, China) as internal standard reference materials were used to monitor the efficiency and accuracy of ICP-MS analysis. Each batch of samples contained at least two blanks and two standard reference materials. Meanwhile, 12.5% of the samples in each batch were randomly selected for parallel sample testing, and the relative standard deviation of parallel samples was <10%. Limits of detection were 0.0003 μg/L for Ce, and recovery of standard rare earth elements (accuracy) ranged from 89.1 to 92%.

### Statistical Analysis

Chi-square tests or Fisher's exact tests were used to compare the distribution of demographic characteristics and environmental factors. The difference of serum Ce between cases and controls was assessed by the Wilcoxon rank-sum test and shown by violin charts. Associations between serum Ce and oral cancer were assessed by calculating odds ratios (ORs) and 95% confidence intervals (95%CIs) using unconditional logistic regression models. Moreover, stratified analyses and interaction terms were used to test the potential modification effects of environmental factors on the level of serum Ce for oral cancer. Additionally, the method proposed by Katsouyanni et al. (15) was utilized to develop a prediction model for oral cancer risk. The predictive ability of the models was evaluated using area under the receiver operating characteristic (ROC) curve (AUC). All tests were based on a 2-sided *P* < 0.05 as evidence of statistical significance. Data analysis was performed using R software version 3.6.0 or GraphPad prism8.0.

## Results

Table 1 shows the main characteristics of the 324 cases and 650 controls. The distributions of age, gender, race, marital status, tobacco smoking and intake of eggs were similar between the case and control groups (*P* > 0.05). However, significant differences were observed with regard to education levels, residence, family history of cancer, alcohol drinking and intake of fish, seafood, leafy vegetables and red meat (all *P* < 0.05).

**Table 1:** Distribution of selected characteristics among patients with oral cancer and controls.

**Variables**	**Cases (%) (*n* = 324)**	**Controls (%) (*n* = 650)**	**χ^**2**^**	***P*-value**
Age(years)			0.41	0.524
<60	138 (42.59)	263 (40.46)		
≥60	186 (57.41)	387 (59.54)		
Gender			2.14	0.143
Male	202 (62.35)	436 (67.08)		
Female	122 (37.65)	214 (32.92)		
Race^a^				0.340
Han	321 (99.07)	648 (99.69)		
Others	3 (0.93)	2 (0.31)		
Education level			15.70	<0.001
Illiterate	32 (9.88)	90 (13.85)		
Primary and middle school	193 (59.56)	433 (66.61)		
High school and above	99 (30.56)	127 (19.54)		
Marital status			1.83	0.176
Married	300 (92.59)	616 (94.77)		
Others	24 (7.41)	34 (5.23)		
Residence			121.95	<0.001
Rural	164 (50.62)	546 (84.00)		
Urban	160 (49.38)	104 (16.00)		
Family history of cancer			9.00	0.003
No	275 (84.88)	593 (91.23)		
Yes	49 (15.12)	57 (8.77)		
Tobacco smoking			3.23	0.072
No	182 (56.17)	404 (62.15)		
Yes	142 (43.83)	246 (37.85)		
Alcohol drinking			15.19	<0.001
No	206 (63.58)	491 (75.54)		
Yes	118 (36.42)	159 (24.46)		
Leafy vegetables (per day)			28.00	<0.001
<2 times	139 (42.90)	170 (26.15)		
≥2 times	185 (57.10)	480 (73.85)		
Red meat (per week)			7.09	0.008
<3 times	96 (29.63)	142 (21.85)		
≥3 times	228 (70.37)	508 (78.15)		
Fish (per week)			27.38	<0.001
<3 times	185 (57.10)	256 (39.38)		
≥3 times	139 (42.90)	394 (60.62)		
Seafood (per week)			17.07	<0.001
<1 time	182 (56.17)	274 (42.15)		
≥1 time	142 (43.83)	376 (57.85)		
Eggs (per week)			2.97	0.085
<3 times	167 (51.54)	297 (45.69)		
≥3 times	157 (48.46)	353 (54.31)		

a *Fisher's exact test*.

**Figure 1:**
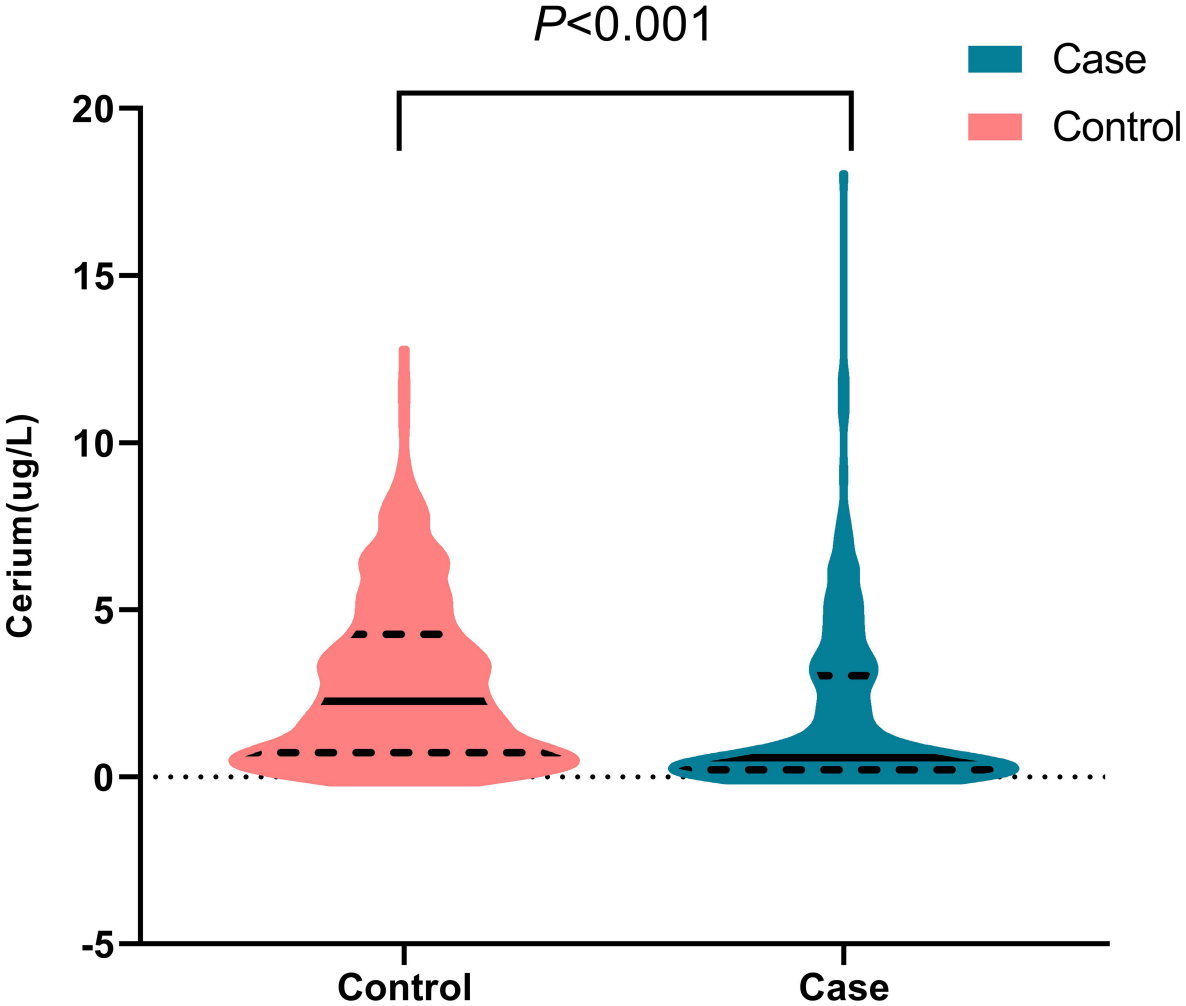
Distribution of serum Ce in case and control groups.

As is shown in Figure 1, the median (quartile25-quartile75) of the concentrations of serum Ce in the oral cancer patients and controls were 0.57(0.21–3.02) μg/L and 2.27(0.72–4.26) μg/L, respectively. Moreover, serum Ce was significantly different between oral cancer patients and controls (*P* < 0.001). When serum Ce was regarded as a continuous variable, an inverse association was observed between serum Ce and the risk of oral cancer [the *OR* was 0.90 (95% *CI*: 0.84, 0.96) for a one-unit increase]. Then, when serum Ce levels were dichotomized into high and low levels, using the median of the serum Ce concentrations in the control group as the cutoffs, high levels of serum Ce was associated with a decreased risk of oral cancer after other potential confounders were controlled (*OR*: 0.60, 95% *CI*: 0.43–0.84, Table 2).

**Table 2:** ORs for the serum levels of Ce in relation to oral cancer.

**Variables**	**Controls (%)**	**Cases (%)**	***OR* (95%*CI*)**	***OR (95%CI***)**^a^**
Ce (Continuous)	650 (100.00)	324 (100.00)	0.83 (0.77–0.88)	0.90 (0.84–0.96)
**Ce(μg/L)**
<2.27(Low)	325 (50.00)	225 (69.44)	1.00	1.00
≥2.27(High)	325 (50.00)	99 (30.56)	0.44 (0.33–0.58)	0.60 (0.43–0.84)

a *Adjustment for age, gender, race, education level, marital status, family history of cancer, residence, tobacco smoking, alcohol drinking, and diet containing leafy vegetables, red meat, fish, eggs and seafood*.

**Figure 2:**
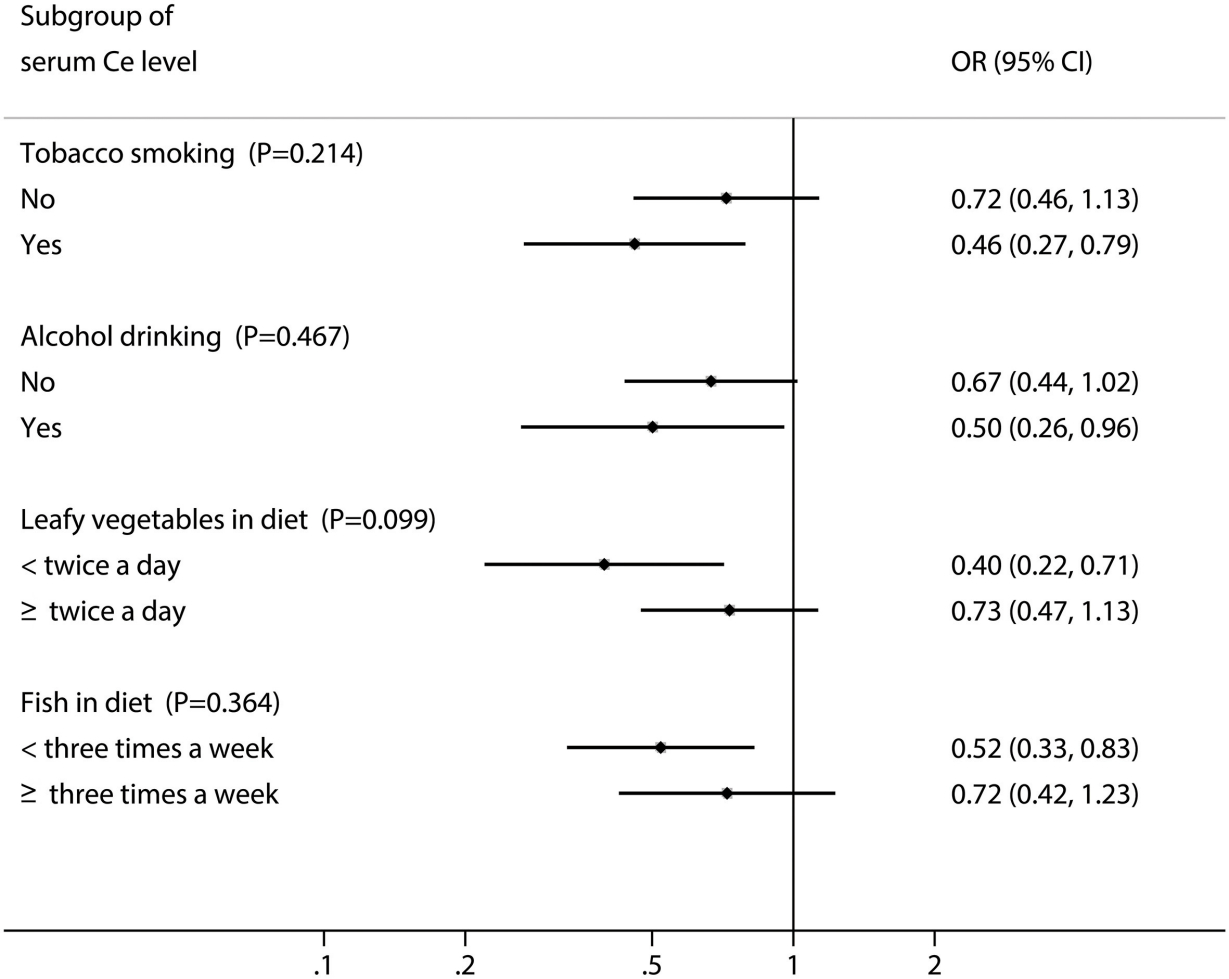
Odds ratios (ORs) and 95% CIs for the serum Ce stratified by environmental factors.

When further stratified by environmental factors, as displayed by Figure 2, stronger inverse associations were observed between the high levels of serum Ce and oral cancer risk among those with smoking (*OR*: 0.46, 95% *CI*: 0.27–0.79), drinking (*OR*: 0.50, 95% *CI*: 0.26–0.96), limited intake of leafy vegetables (*OR*: 0.40, 95% *CI*: 0.22–0.71) and fish (*OR*: 0.52, 95% *CI*: 0.33–0.83). Additionally, there were significant multiplicative interactions between the serum levels of Ce and alcohol drinking or intake of leafy vegetables and fish for oral cancer (all *P*_interaction_ <0.05, Table 3).

**Table 3:** Combined effect of the serum levels of Ce and tobacco smoking, alcohol drinking or intake of leafy vegetables or fish for oral cancer.

**Variables**		**Case (%)**	**Control (%)**	**Adjusted *OR* (95% *CI*)^a^**	***P*_**interaction**_**
Ce (μg/L)	Tobacco smoking				
<2.27(Low)	Yes	105 (28.85)	117 (13.93)	1.00	
<2.27(Low)	No	120 (32.97)	208 (24.76)	0.49 (0.30–0.79)	0.003
≥2.27(High)	Yes	37 (10.16)	129 (15.36)	0.42 (0.25–0.70)	0.001
≥2.27(High)	No	62 (17.03)	196 (23.33)	0.38 (0.22–0.65)	<0.001
Ce × Tobacco smoking	–	–	0.70 (0.47–1.04)	0.081
Ce (μg/L)	Alcohol drinking				
<2.27(Low)	Yes	89 (24.45)	90 (10.71)	1.00	
<2.27(Low)	No	136 (37.36)	235 (27.98)	0.44 (0.28–0.70)	0.001
≥2.27(High)	Yes	29 (7.97)	69 (8.21)	0.47 (0.26–0.86)	0.014
≥2.27(High)	No	70 (19.23)	256 (30.48)	0.30 (0.18–0.49)	<0.001
Ce × Alcohol drinking	–	–	0.56 (0.39–0.81)	0.002
Ce (μg/L)	Leafy vegetables (per day)				
<2.27(Low)	<2 times	103 (28.30)	90 (10.71)	1.00	
<2.27(Low)	≥2 times	122 (33.52)	235 (27.98)	0.46 (0.31–0.69)	<0.001
≥2.27(High)	<2 times	36 (9.89)	80 (9.52)	0.52 (0.30–0.91)	0.022
≥2.27(High)	≥2 times	63 (17.31)	245 (29.17)	0.30 (0.19–0.48)	<0.001
Ce × Leafy vegetables	–	–	0.51 (0.35–0.74)	<0.001
Ce (μg/L)	Fish (per week)				
<2.27(Low)	<3 times	127 (34.89)	114 (13.57)	1.00	
<2.27(Low)	≥3 times	98 (26.92)	211 (25.12)	0.37 (0.24–0.57)	<0.001
≥2.27(High)	<3 times	58 (15.93)	142 (16.90)	0.46 (0.29–0.71)	0.001
≥2.27(High)	≥3 times	41 (11.26)	183 (21.79)	0.32 (0.20–0.51)	<0.001
Ce × Fish	–	–	0.57 (0.37–0.86)	0.008

a *Adjustment for age, gender, race, education level, marital status, family history of cancer, residence, tobacco smoking, alcohol drinking, and diet containing leafy vegetables, red meat, fish, eggs and seafood*.

**Figure 3:**
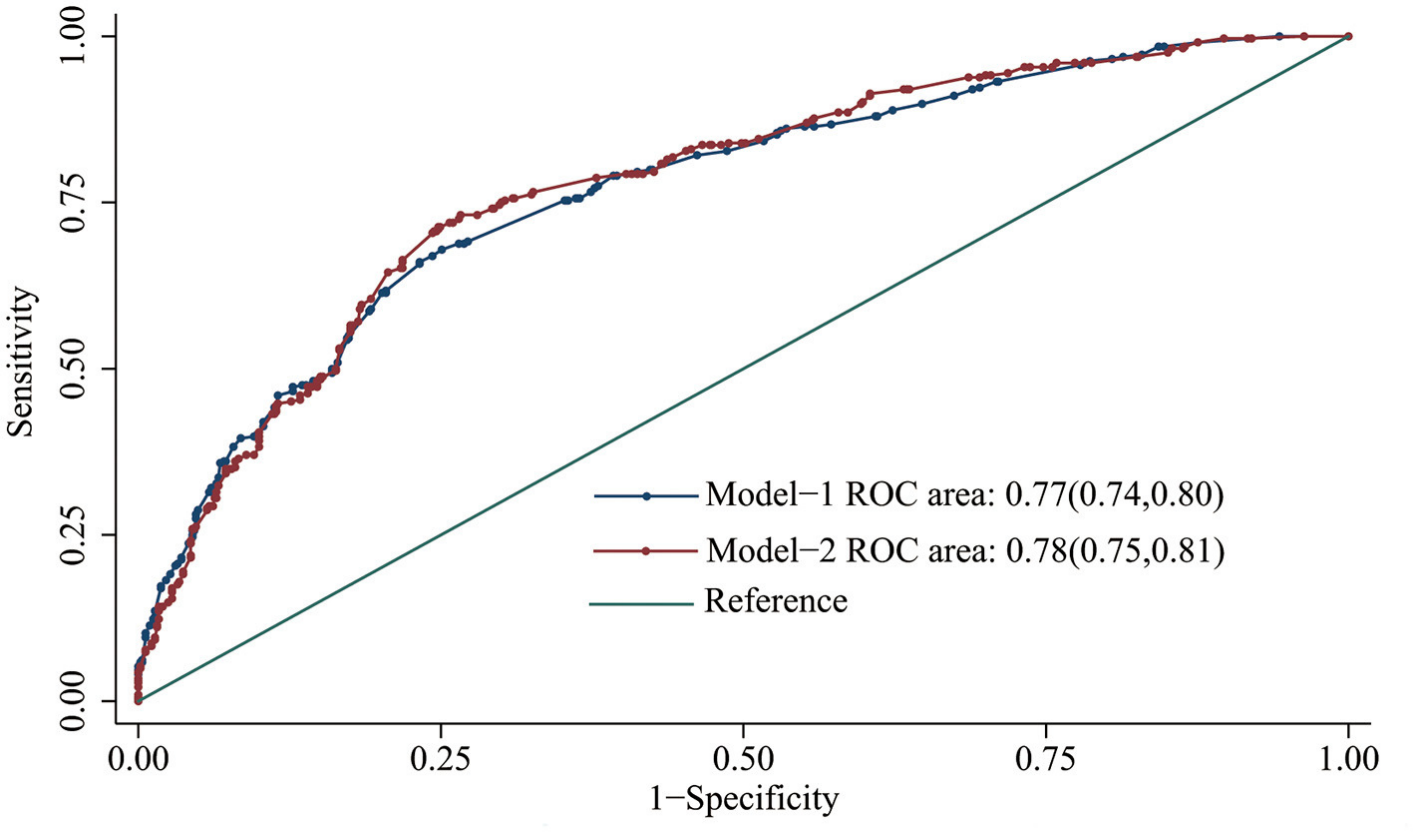
Receiver operating characteristic curve of different multifactor models for predicting oral cancer risk.

Subsequently, a multivariate logistic regression analysis was performed to develop different models by incorporating serum Ce and environmental factors (Table 4). Compared with model-1, model-2 had the higher discriminatory ability for oral cancer risk, with the lower Akaike information criterion (AIC) value (1033.82). Moreover, the area under the curve of model-2 was larger than that of model-1 (*P* = 0.015, Figure 3).

**Table 4:** Multivariate unconditional logistic regression analysis of influencing factors of oral cancer.

**Variables**	**Model-1^a^**		**Model-2^b^**	
	***P***	***OR* (95%*CI*)**	***P***	***OR* (95%*CI*)**
**Tobacco smoking**				
No		1.00		1.00
Yes	0.018	1.64 (1.09–2.46)	0.019	1.64 (1.09–2.48)
**Alcohol drinking**				
No		1.00		1.00
Yes	<0.001	2.10 (1.42–3.11)	<0.001	2.03 (1.37–3.01)
**Leafy vegetables (per day)**				
<2 times		1.00		1.00
≥2 times	<0.001	0.49 (0.36–0.68)	<0.001	0.50 (0.36–0.69)
**Red meat (per week)**				
<3 times		1.00		1.00
≥3 times	0.097	0.73 (0.51–1.06)	0.072	0.71 (0.49–1.03)
**Fish (per week)**				
<3 times		1.00		1.00
≥3 times	<0.001	0.48 (0.34–0.67)	<0.001	0.48 (0.35–0.67)
**Seafood (per week)**				
<1 time		1.00		1.00
≥1 time	0.002	0.59 (0.42–0.83)	0.002	0.58 (0.41–0.82)
**Eggs (per week)**				
<3 times		1.00		1.00
≥3 times	0.257	0.83 (0.61–1.14)	0.220	0.82 (0.60–1.13)
**Ce(μg/L)**				
<2.27 (Low)		–		1.00
≥2.27 (High)	–	–	0.003	0.60 (0.42–0.84)
AIC		1040.50		1033.82

a *Adjustment for age, gender, race, education level, marital status, family history of cancer, residence*.

b *Additional adjustment of serum Ce*.

## Discussion

In this relatively large-scale case-control study, we systematically evaluated the serum Ce and risk of oral cancer using an advanced analytical technique (ICP-MS). The results revealed that high serum Ce was significantly associated with decreased risk of oral cancer. Interestingly, the inverse association was more obvious among tobacco smokers, alcohol drinkers or those consumed fewer leafy vegetables or fish. Additionally, significant multiplicative interactions between serum levels of Ce and alcohol drinking or intake of leafy vegetables or fish for oral cancer were also observed.

Several studies have revealed the anticancer activity of Ce in many types of cancer cells. Giri et al. (12) showed that increase of nanoceria expression was associated with the increased ability to inhibit metastasis and angiogenesis in ovarian cancer cells, thus contributing to attenuate ovarian tumor growth (12). Moreover, Ce was also found to exhibit toxicity in human colon cancer cells, neuroblastoma cells, and fibrosarcoma cells through generating ROS which induces apoptotic cell death (11, 16, 17). We speculated that the association of high serum Ce levels with decreased risk of oral cancer may be attributed to the anticancer potential of Ce in cancer cells. Because of the increased rate of glycolysis and lactic acid production, cancer cells develop relatively a higher acidic environment than healthy cells. The lower pH environment causes loss of antioxidant (cytoprotective) ability of Ce, instead it behaves as a pro-oxidant, which may facilitate the production of ROS to cause tumor cell apoptosis (18, 19). Interestingly, when stratified by smoking status, the protective effect of high levels of serum Ce in smokers on oral cancer was higher than that in non-smokers. It was well-established that a large number of free radicals in cigarette smoke attacked the genetic material of cells directly or indirectly, and played a crucial role in the process of inducing and promoting cancer (20). Whereas, Ce could prevent the formation of free radicals and ROS by mimicking the functions of antioxidant enzymes such as superoxide dismutase and catalase (21). A possible mechanism for this due to the fact that high levels of serum Ce can interfere with the production of smoking-induced free radicals through antioxidation. Further research is still needed to explore the exact mechanism.

Moreover, our data revealed that the association between serum Ce levels and the risk of oral cancer was modified by alcohol drinking, with multiplicative interactions between them. Alcohol intake was found to reduce the immune function of the body, and caused the increased permeability of oral mucosa to carcinogens (22, 23). Data from a chronic toxicity test indicated that the oral administration of Ce at low and moderate doses could significantly improve the immunity of male rats by increasing white blood cells (WBCs) and the total lymphocyte counts (24). Therefore, a possible explanation is that Ce may reduce the risk of oral cancer caused by alcohol through strengthening the immune system of the human body.

Additionally, the result showed that there was a multiplicative interaction between serum cerium and green leafy vegetable intake, and the protective effect was the strongest in the group of high serum cerium level and high vegetable intake. On the one hand, some studies have confirmed that a diet adequate in vegetables may protect against oral cancer (25, 26). Although the mechanisms by which vegetables may confer protection against cancer is not entirely clear, cruciferous vegetables are important sources of glucosinolates, whose major breakdown products (isothiocyanates and indoles) have been demonstrated to have anti-carcinogenic properties *in vitro* as well as in animal studies (27, 28). On the other hand, cerium has the effect of antioxidation. Therefore, serum cerium and adequate vegetable intake play a synergistic role in the pathogenesis of oral cancer.

There are also limitations of the current study that merit mention. First, we only collect blood samples at the first visit, and the exposure time reflected might not be enough to cover the latency of oral cancer development (29), which precluded us from firmly establishing a temporal association between serum Ce and oral cancer. Therefore, although we hypothesized that the concentration of Ce measured in our study reflected the level of pre-cancerous cancer, we cannot rule out the possibility that the concentration is affected by oral cancer. Consequently, further prospective studies are needed to confirm our findings. Second, Ce can be present in some solid or solution in the form of a positive divalent, tetravalent, or mixed valence (30). Nevertheless, the detection methods used in this study cannot determine the valence state of Ce, hopefully, which could be distinguished in future studies.

## Conclusion

In conclusion, this preliminary case-control study reveals lower serum Ce levels in patients with oral cancer than that of healthy controls, and suggests an inverse association between high serum Ce levels on the risk of oral cancer. This association may be modified by smoking, alcohol drinking, and dietary patterns. Much more extensive research is warranted to confirm these possible associations and to clarify their mechanisms, which will promote the application of Ce in the diagnosis and treatment of oral cancer and provide a new strategy for improving tumor prevention.

## Data Availability Statement

The raw data supporting the conclusions of this article will be made available by the authors, without undue reservation.

## Ethics Statement

The studies involving human participants were reviewed and approved by the Ethics Committee of Fujian Medical University. The patients/participants provided their written informed consent to participate in this study.

## Author Contributions

FC and BH participated in the design of the study. LL, BS, YQ, ZZ, and YH were responsible for recruitment and interview participants. LP and XZ contributed to samples collection. JW, JC, and JL performed laboratory experiments. JL, FL, and LY analyzed the data. BH, JW, and FC wrote the manuscript, which was revised by all authors. All authors contributed to the article and approved the submitted version.

## Conflict of Interest

The authors declare that the research was conducted in the absence of any commercial or financial relationships that could be construed as a potential conflict of interest.
